# Proteomic Analysis of Hsp90β-Selective Inhibitors Against Triple-Negative Breast Cancer to Gain a Mechanistic Insight

**DOI:** 10.1016/j.mcpro.2025.101043

**Published:** 2025-07-24

**Authors:** Tyelor S. Reynolds, Daniel D. Hu, Simon D. Weaver, Emma C. Ronck, Sanket J. Mishra, Matthew M. Champion, Brian S.J. Blagg

**Affiliations:** 1 Department of Chemistry and Biochemistry, The University of Notre Dame, Notre Dame, Indiana, USA; 2 Grannus Therapeutics Inc, South Bend, Indiana, USA

**Keywords:** Hsp90β, TNBC, Hsp90, proteomics, client substrates

## Abstract

Hsp90 (90 kDa heat shock protein) is a central molecular chaperone responsible for the folding and activation of >400 client proteins, causing it to be a highly sought after drug target for the treatment of cancer. Hsp90 pan-inhibitors have been evaluated in the clinic, but on- and off-target toxicities have limited their development. The emergence of Hsp90β-selective inhibitors has been proposed as a safer therapeutic alternative. However, since they in theory only target a portion of the Hsp90-regulated proteome, a deeper understanding of their mechanism of action and whether they exhibit selectivity for cancer over normal cells has remained uninvestigated. Herein, we show that Hsp90β-selective inhibitors, NDNB1 and NDNB1182, exhibit a moderate selectivity for triple-negative breast cancer (TNBC) over normalized MCF-10A cells in contrast to pan-inhibitors, which do not exhibit a selectivity. This article contains the first proteomic analysis of Hsp90β-selective inhibitors. We have employed a traditional bottom–up LC–MS/MS proteomics approach to explore the potential mechanisms of action underlying the anticancer effects of NDNB1 and NDNB1182 against TNBC. Primarily, inhibition of kinases and associated cell signaling pathways, cell cycle proteins, and DNA repair were notable processes affected by Hsp90β inhibition, to name a few. Further investigation of the impact on Hsp90β interactors allowed a fuller understanding of Hsp90β-dependent processes. We also identified RAD9A, cyclin-dependent kinase 1 (CDK1), and ribosomal protein S9 (RPS9) as potential Hsp90β client substrates. The three example proteins selected exemplify a mechanistic explanation for inhibition of DNA repair (RAD9A), cell cycle (CDK1), and translation (RPS9), shedding some light on some of the implications of Hsp90β inhibition as it pertains to TNBC. Therefore, previously unknown clients, Hsp90β interactors, or Hsp90β-regulated proteins could be determined using the results from this study.

## Highlights

• Bottom–up proteomics approach to study Hsp90β-selective inhibitors.
• Hsp90β-selective inhibitors are effective against triple-negative breast cancer.
• Hsp90β inhibition disrupts kinases, the cell cycle, and DNA repair.
• RAD9A, CDK1, and RPS9 are potential Hsp90β-dependent clients.

## Graphical Abstract

**Figure:**
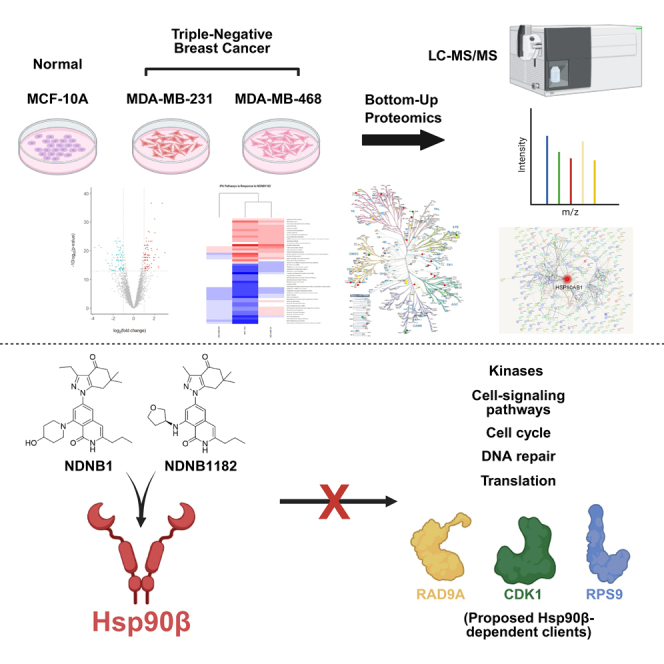

## In Brief

A traditional bottom–up LC–MS/MS proteomics approach was employed to explore the potential mechanisms of action underlying the anticancer effects of NDNB1 and NDNB1182 against triple-negative breast cancer. Inhibition of kinases and associated cell signaling pathways, cell cycle proteins, and DNA repair were notable processes affected by 90 kDa heat shock protein (Hsp90β) inhibition, to name a few. Further investigation of the impact on Hsp90β interactors allowed a fuller understanding of Hsp90β-dependent processes. We also identified RAD9A, CDK1, and RPS9 as potential Hsp90β client substrates.

## Introduction

Members of the 90 kDa heat shock protein (Hsp90) family are ubiquitously expressed and structurally conserved molecular chaperones (1, 2). The Hsp90 family consists of two cytosolic isoforms, inducible Hsp90α and the constitutively expressed Hsp90β (2). In addition, glucose-regulated protein 94 (Grp94) resides in the endoplasmic reticulum, and mitochondrial tumor necrosis factor receptor–associated protein-1 (Trap1) is localized to the mitochondria (3, 4). Together, the four Hsp90 isoforms maintain protein homeostasis in response to cellular stresses and regulate the stability and activity of >400 client proteins. Hsp90 has been evaluated as a promising therapeutic target for cancer, since it represents a single molecular target that can simultaneously modulate several oncogenic pathways (5). In fact, Hsp90’s diverse array of client proteins are associated with the progression of all 10 hallmarks of cancer (6, 7). Twenty-two small-molecule Hsp90 pan-inhibitors have entered clinical trials for the treatment of cancer; however, complications have arisen, causing many to fail (5). Many of these complications, such as induction of the heat shock response and concerns with on-target cardiotoxicity and oculartoxicity, have been attributed to the inhibition of Hsp90α. As an alternative strategy, recent developments have been aimed at the design of Hsp90 isoform–selective inhibitors to overcome the previously faced detriments by avoiding Hsp90α inhibition (8, 9, 10, 11, 12).

Hsp90α is the inducible isoform and is upregulated in response to a variety of cellular insults to assist in the adaptation to stress, which is why cancers commonly overexpress levels of Hsp90α (13, 14). In contrast, Hsp90β is constitutively expressed and acts as a “housekeeping” chaperone to ensure the essential functionality of the cell (15). Consequently, Hsp90β is essential for cellular viability, whereas Hsp90α is only essential during spermatogenesis (16, 17, 18). Although much of the original rationale that made Hsp90 a promising drug target was directed at Hsp90α′s role in aiding cancer progression, Hsp90α and Hsp90β share a significant overlap of client proteins, which appear to serve an intrinsic redundancy. In fact, they share 183 clients, whereas Hsp90α has 135 unique clients and Hsp90β has 410 unique clients (15). Examples of shared biological processes (BPs) between the two cytosolic isoforms include response to stimuli, protein phosphorylation, and cell communication. However, Hsp90α and Hsp90β still maintain distinct functions. For example, Hsp90β regulates cyclin-dependent kinase (CDK4) and CDK6 stability, which are responsible for cell cycle phase transition. In addition, protein ubiquitination, cytoskeleton organization, and protein catabolism were identified as BPs uniquely associated with Hsp90β clients (15).

While inhibition of an essential, ubiquitously expressed protein warrants reservation, there is evidence to support that Hsp90 inhibitors exhibit a selectivity for cancer over normal cells: The active Hsp90 heteroprotein complex exhibits ∼200-fold higher affinity for ATP than the Hsp90 homodimer found in normal tissue, providing rationale for cancer selectivity (19). It has also been observed that Hsp90 inhibitors preferentially accumulate in tumors (20, 21). A structure-based molecular design and optimization approach was employed by our laboratory to develop ATP-competitive Hsp90β-selective inhibitors, which bind at the N-terminal ATP-binding site (9, 22). Two of the lead Hsp90β-selective inhibitors at the time of this study were NDNB1182, which exhibits an affinity of 65 nM for Hsp90β (>153-fold selective over Hsp90**a**) and NDNB1 (225 nM for Hsp90β, 333-fold selective over Hsp90**a**). NDNB1182 was shown to enhance immune checkpoint blockade (ICB) therapy *in vivo*, resulting in a significant reduction in tumor volume and an increase in survival rate without significant effects on body weight (23). Furthermore, NDNB1182 treatment manifested no discernable histological differences in the liver or the spleen, suggesting that efficacy is achievable at nontoxic doses. Recent work showed that Hsp90β-selective inhibitors avoid disruption of cardiomyocyte function and toxicity to both cardiomyocytes and human retinal cells, in contrast to Hsp90 pan-inhibitors (*e.g.*, 17-AAG).

The exact mechanisms underlying the effectiveness of Hsp90β-selective inhibitors remain unknown, but they appear to be effective against triple-negative breast cancer (TNBC) and other select cancers. No studies to date have investigated the effects of Hsp90β-selective inhibitors on the proteome. Previous proteomics studies were performed utilizing pan-Hsp90 inhibitors such as 17-DMAG (24). Sharma *et al.* investigated the pathways differentially affected by 17-DMAG and particularly emphasized 17-DMAG’s impact on kinases. A similar systematic study was conducted using the pan-Hsp90 inhibitor geldanamycin, which also investigated the kinetics of Hsp90 client degradation (25). Other studies have also been performed, including protein ubiquitinylation and the use of thermal profiling, to name a few (26, 27, 28, 29, 30). However, the use of pan-Hsp90 inhibitors has at best allowed the determination of both Hsp90α- and Hsp90β-dependent effects. Therefore, the purpose of this study was to provide an understanding of how Hsp90β-selective inhibitors manifest anticancer effects by examining the proteomic data of both a normal breast and TNBC cell lines.

## Experimental Procedures

### Cell Culture

MCF-10A, MDA-MB-231, and MDA-MB-468 cells were grown in a water-jacketed incubator at 37 °C with 5% CO_2_. MDA-MB-231 and MDA-MB-468 cells were cultured in Dulbecco's modified Eagle's medium (Corning; catalog no.: 10-013-CV) supplemented with 10% heat-inactivated FBS (Gibco; catalog no.: 10438-026) and 1% penicillin–streptomycin (VWR; catalog no.: K952-100ML). MCF-10A cells were cultured in Dulbecco's modified Eagle's medium/F-12K (Gibco; catalog no.: 10565018) + GluMAX media supplemented with 5% horse serum (Gibco; catalog no.: 16050130), 1% penicillin–streptomycin, 20 ng/ml epidermal growth factor (Gibco; catalog no.: PHG0311), 0.5 mg/ml hydrocortisone (Thermo; catalog no.: A16292-03), 100 ng/ml cholera toxin (Sigma; catalog no.: C8052), and 10 μg/ml insulin (Gibco; catalog no.: 12585014).

### MTS Growth Assay

MCF-10A, MDA-MB-231, or MDA-MB-468 cells were plated in clear flat-bottom 96-well plates (VWR; catalog no.: 10861-666) at a density of 3000 cells per well. The next day, cells were treated either with 1% dimethyl sulfoxide (DMSO; vehicle control) or compound. Cell viability was determined after 72 h using CellTiter 96 Aqueous One Solution Cell Proliferation assay (Promega Corp). CellTiter 96 solution (20 μl) was added to each well, and the plates were incubated for an additional 3 h at 37 °C, after which the absorbance of each well was measured at 490 nm using a microplate spectrophotometer (BioTek Epoch). All assay wells were performed in triplicate, and each assay was repeated three times. Cell growth of treated plates was calculated relative to an untreated time 0 plate measured at the time of treatment. IC_50_ values are reported as the mean ± SD of N = 3 biological replicates. Statistical analysis was performed using GraphPad Prism V9 (GraphPad Software, Inc).

### Western Blot Analysis

Cells were seeded at 300,000 cells/well in 6-well plates (VWR; 10861-696). Once cells had reached ∼80% confluency, the media were aspirated and replaced with 2 ml of media containing compound or vehicle (0.25% DMSO) and incubated for a 24-h treatment time. After 24 h, cells were washed with ice-cold PBS and then lysed with cell lysis buffer (130 mM NaCl, 1% Triton X-100, 1 mM EDTA, 0.1% SDS, 10 mM Tris–Cl [pH 8.0] in water + freshly added 1 mM Protease Cocktail 2, 1 mM Protease Cocktail 3, 1 mM phosphatase inhibitor, and 1 mM PMSF). Cell lysates were obtained by centrifugation at 10, 000 rpm for 10 min at 4 °C. Protein concentrations were determined using the Pierce Bicinchoninic Acid assay kit following the manufacturer's instructions. Then, 20 μg of each normalized protein lysate was electrophoresed on 10% SDS-polyacrylamide gels and transferred onto polyvinylidene difluoride membranes. Membranes were washed in Tris-buffered saline containing Tween (10 mM Tris–HCl, 150 mM NaCl, pH 7.2, and 0.1% Tween-20), incubated in blocking buffer (7% nonfat milk in H_2_O), and then incubated with primary antibodies at 4 °C overnight. After washing with Tris-buffered saline containing Tween, membranes were incubated in their respective secondary antibodies for 1 h at room temperature. The blots were developed using Clarity Max Western ECL Blotting Substrates (Bio-Rad). The following primary antibodies were obtained from Cell Signaling Technology: CXCR4 (D4Z7W), cIAP-1 (D5G9), Akt (9272), β-Actin (8H10D10), P53 (7F5), CDK4 (D9G3E), CDK6 (DCS83), and Survivin (71G4B7). The following primary antibodies were obtained from Enzo Life Sciences: Hsp70/Hsp72 (C92F3A-5) and Hsp90α (9D2). All primary antibodies were used at 1:1000 dilutions, and all secondary antibodies were used at 1:2000 dilutions unless otherwise stated. Horseradish peroxidase–conjugated secondary goat anti-mouse IgG, goat anti-rat IgG, and goat anti-rabbit IgG antibodies were purchased from Southern Biotech.

### Materials

LC–MS-grade water, acetonitrile, acetone, and methanol were from JT Baker. Tris(2-carboxyethyl)phosphine was from Thermo Scientific. Iodoacetamide and SDS were from VWR. Triethylammonium bicarbonate (TEAB) and phosphoric acid were from Sigma–Aldrich. Trypsin was from Promega. S-Trap Minis were from ProtiFi. Hydrophilic–lipophilic balance solid-phase extraction (SPE) cartridges were from Waters. LC–MS/MS system used is a nanoElute 2 and timsTOF Pro 2 from Bruker Scientific.

### Experimental Design and Statistical Rationale

#### Sample Preparation

Five biological replicates of each treatment were prepared for each cell line (DMSO, NDNB1, and NDNB1182). Cell lysates were prepared following the same aforementioned procedure and in the study by Hu *et al.* (31, 32). Protein concentration was determined by bicinchoninic acid assay, and the samples were normalized to 100 μg in 100 μl and stored at −80 °C until further use. Each sample had 100 μg equivalent volume of lysate portioned in aliquots. Samples were vacuum concentrated to ≤60 μl. The sample had cold acetone added to a final volume of 1300 μl, incubated for 1 h at −20 °C, and centrifuged at 12,000*g* for 10 min to pellet proteins. Acetone was decanted, and samples were concentrated to dryness. Samples were resuspended in 10 μl 100 mM TEAB, 5 μl 1M TEAB, and 25 μl 20% SDS, with frequent heating and vortexing to bring proteins into solution. Samples were reduced with 5 μl 100 mM TEAB at 95 °C for 10 min, then alkylated with 5 μl 100 mM iodoacetamide in darkness for 30 min. Samples were acidified with 5 μl 12% H_3_PO_4_, flocculated with 350 μl 100 mM TEAB in 90% methanol, and loaded onto S-Trap Mini spin columns. Three wash steps were performed by adding and centrifuging two 150 μl additions of 100 mM TEAB in 90% methanol and one 150 μl addition of 50% methanol and 50% chloroform. Spin columns were put in new collection tubes, and 2 μg of trypsin in 160 μl 100 mM TEAB was added to each filter column. Samples were incubated at 37 °C overnight, then spun down and eluted in two additions of 80 μl 0.1% formic acid, and one addition of 80 μl 0.1% formic acid in 50% acetonitrile. Samples were vacuum concentrated for 20 min at 50 °C to evaporate residual acetonitrile from elution, in preparation for desalting.

Samples were desalted using Oasis hydrophilic–lipophilic balance SPE cartridges on a vacuum manifold. Cartridges were wet with two additions of 500 μl acetonitrile and then equilibrated in three additions of 0.1% formic acid in water. Samples were loaded onto individual cartridges, and two washes of 250 μl 0.1% formic acid in water were performed. SPE cartridges were removed, placed over new microcentrifuge tubes, and three elutions of 200 μl 0.1% formic acid in 50% acetonitrile were performed. Samples were vacuum concentrated to dryness, followed by resuspension in 0.1% formic acid to 1 mg/ml.

### LC–MS/MS Analysis

Samples were analyzed on a nanoElute 2 and timsTOF Pro 2 LC–MS/MS system with CaptiveSpray (20 μm emitter). Each sample (1 μg) was injected onto a PepSep XTREME C18 column (25 cm × 150 μm × 1.5 μm) at 1.2 μl/min flow rate. About 120 min gradients (5%–35% B) were performed in technical triplicate. Data-independent acquisition parallel accumulation-serial fragmentation (DIA-PASEF)was performed on manufacturer’s default settings with mass windows drawn using heatmap from a data-dependent acquisition-parallel accumulation-serial fragmentation run acquired on one sample (x01). Windows were drawn to occupy the greatest amount of ions (excluding +1 range) with a mass width of 26.0 Da, mass overlap of 1.0 Da, and a cycle time estimate of under 3.0 s. Each biological replicate was analyzed in technical triplicate injections. Raw files can be found on MassIVE with identifier MSV00096357 (ftp://MSV00096357@massive.ucsd.edu) and Proteome Exchange PXD (PXD057678).

### Database Searching

Raw files were searched using Spectronaut (18.7.240506.55695) using directDIA+ analysis and the human proteome database downloaded from UniProt on 2-6-2024 (UP000005640_9606) containing 20,598 entries. Each cell line was searched as a separate experiment with the default direct DIA settings including two allowed missed cleavages, false discovery rates of 0.01 at the peptide and protein level, and the following modifications: Fixed—carbamidomethyl (C); variable—acetyl (protein N-term), deamidation (NQ), Gln-> pyro-Glu, Glu -> pyro-Gly, Oxidation (M). The default mass tolerance settings for directDIA+ searches were used, which includes dynamic tolerances calculated per run during calibration that scale over retention time and ion mobility. For this experiment, average mass tolerances ranged from 9.7 ppm to 13.8 ppm for MS1 and 9.5 ppm to 13.6 ppm for MS2. Full search settings can be found in the supplemental data. The results were exported as *.tsv* files for each search with the protein pivot report including additional information about the number of precursors identified for each protein in each injection.

### Data Analysis

Data exports were further analyzed in R (v4.3.1), using the packages QFeatures (v1.10.0) and limma (v3.56.2) using a workflow modified from Hutchings *et al*. (33, 34, 35) Protein quantities were filtered so that any injection that was quantified with only a single precursor was set to NA (minimum two peptides required). Protein quantities were log2 transformed and normalized with the *center.median* method from QFeatures. All the technical replicates for a single biological replicate were averaged to create a single quantity for each protein. These quantities were plotted for each pairwise comparison (WT *versus* treatment), and a quadratic fit was applied for normalization to a linear correlation. Limma was used to perform differential expression analysis by creating a linear model fit with the *lmFit()* function, and empirical Bayes statistics were calculated with *eBayes()*, which includes a Benjamini–Hochberg (B–H) correction for multiple hypothesis testing to produce adjusted *p* values (36). 95% Confidence intervals were calculated from these adjusted *p* values (37). Differential expression plots were created by plotting the average expression of each protein *versus* log_2_ fold change (LFC) between treatment and WT. Proteins with LFC >1 and an adjusted *p* value of <0.05 (adj. *p* < 0.05) were highlighted, with the 95% confidence intervals plotted as error bars. Protein statistics were exported as .csv files for pathway analysis and can be found in the supplemental data. The full scripts for this analysis can be found in github (https://github.com/Champion-Lab/HSP90Binh).

Protein annotation was determined by UniProt accession identifiers and their associated official gene symbols. R statistical programming language (version 4.2.2) in RStudio (version 2023.6.1.524) was used to generate volcano plots with the ggplot2 package (38, 39, 40). Proteins with an adj. *p* < 0.05 and an LFC >1 were colored with downregulated proteins in blue, upregulated proteins in red, and the nonstatistically measured or dysregulated proteins in gray. R ggplot2 and Venn Diagram (version 1.7.3) were used to generate Venn diagrams of proteins that were either identified or differentially regulated in the three cell lines and/or two Hsp90β-selective inhibitors (41).

The top upregulated or downregulated proteins were analyzed by filtering for all proteins that had an LFC >1 and an adj. *p* < 0.05, and then Hsp90β interactors were identified if they had either been reported in The Hsp90 Chaperone Machine Database (Picard) as previously discussed or in The Biological General Repository for Interaction Datasets (BioGRID) https://thebiogrid.org/109558/summary/homo-sapiens/hsp90ab1.html (accessed July 25, 2024) (892 after removing duplicates) (42). Two databases were used to broaden the scope of potential previously identified Hsp90β interactors for the most dysregulated proteins. The proteins commonly upregulated or downregulated across different cell lines or the two inhibitors were also assessed to search for reliable and conserved Hsp90β-regulated proteins.

The LFC between treatment and WT for each cell line was used to perform a meta differential expression between cell lines, using B-H adjusted *t* tests. Volcano plots were created to show the change in LFC and significance for each treatment between cell lines. A similar analysis was performed to compare the two treatments, NDNB1 *versus* NDNB1182, in each cell line, using *t* tests and B–H adjusted significance. In addition, Pearson's correlation coefficients were calculated using R.

### Gene Ontology Enrichment/Pathway Analysis/Interaction Mapping

The Database for Annotation, Visualization and Integrated Discovery (DAVID) Bioinformatics Database was used to perform a Gene Ontology (GO) search enrichment analysis for BPs (GO BP) (43, 44). All proteins with an LFC >1 and either an adj. *p* < 0.05 or adj. *p* < 0.2 cutoff was used. Downregulated and upregulated proteins were searched separately. Sorted by -log_10_(*p* value).

Data were also analyzed with the use of QIAGEN Ingenuity Pathway Analysis (QIAGEN, Inc, https://digitalinsights.qiagen.com/IPA) (45). Proteins with an adj. *p* < 0.05 were used in all samples except in some analyses where an adj. *p* < 0.2 was used for the MDA-MB-231 and MDA-MB-468 samples where it is explicitly stated. Canonical pathway activation was collected from ingenuity pathway analysis (IPA) (B–H adj. *p* < 0.05) and sorted by *z*-score into the top 50 pathways. These lists were then used in R with ggplot2 to generate heat maps.

Hsp90β interactors were selected from The Hsp90 Chaperone Machine Database (Picard, https://www.picard.ch/Hsp90Int/accessed July 15, 2024) by searching for HSP90AB1 (https://www.picard.ch/Hsp90Int (accessed 2024 07-15)). About 737 unique interactors were reported. Proteins that had an LFC >0.5 and an adj. *p* < 0.2 that were identified to be Hsp90β interactors were used in the DAVID GO BP search as aforementioned. An IPA search of all 737 interactors, even those not identified in the dataset, was performed to determine the protein type and localization. All proteins that were detected as kinases after ingenuity pathway analysis were sorted based on whether they were reported as Hsp90β interactors and exhibited an LFC >0.5 and an adj. *p* < 0.05. Then, they were mapped onto a map of the human kinome with KinMap (46). In addition, all kinases from the 737 Hsp90β interactors list (Picard) were mapped as a comparison. A DAVID search of all 737 interactors was completed to perform an enrichment analysis of GO BP, GO molecular function, GO cellular component, and Kyoto Encyclopedia of Genes and Genomes pathways. The results were rank ordered by -log_10_(*p* value).

The Search Tool for the Retrieval of Interacting Genes/Proteins (STRING) database was used to generate functional protein association networks (47). Proteins that had an LFC <−1 and an adj. *p* < 0.05 were searched to visualize potential protein–protein interaction networks.

## Results

**Fig. 1:**
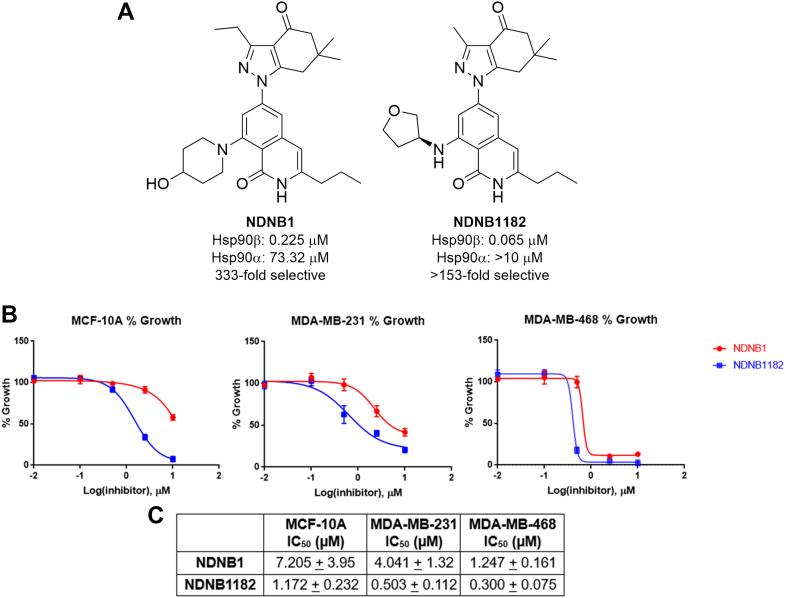
**Structures and inhibitory activities of Hsp90β-slective inhibitors.***A*, previously published lead Hsp90β-selective inhibitors NDNB1 and NDNB1182. *B*, MTS dose–response growth curves after 72 h treatment with NDNB1 or NDNB1182. *C*, respective IC_50_ values reported as the mean ± SD of N = 3 biological replicates.

Hsp90β-selective inhibitors were assessed for their ability to inhibit the growth of an MCF-10A normalized breast cell line and the MDA-MB-231 and MDA-MB-468 TNBC cell lines after 72 h via an MTS cell proliferation assay. NDNB1 and NDNB1182 exhibited low micromolar antiproliferative IC_50_ values and a ∼2-5-fold selectivity for TNBC over MCF-10A cells (Fig. 1). The data support that Hsp90β-selective inhibitors display a cancer selectivity, at least *in vitro*, which appears to be in agreement with the safety profile previously observed (23). Inhibition of Hsp90β was assessed by treating MCF-10A, MDA-MB-231, and MDA-MB-468 cells with NDNB1 and NDNB1182, followed by Western blot analysis (Supplemental Fig. S1). NDNB1 and NDNB1182 caused a dose-dependent degradation of Hsp90β-dependent clients (*e.g.*, CDK4, CDK6, Akt), while avoiding the degradation of the Hsp90α-specific client, Survivin. Degradation of other reported Hsp90β clients, such as CXCR4 and cIAP-1, was less pronounced and required higher concentrations. A consistent dose-dependent induction of Hsp70 was observed in all samples, meanwhile an induction of Hsp90α appeared to be drug and/or cell line dependent.

**Fig. 2:**
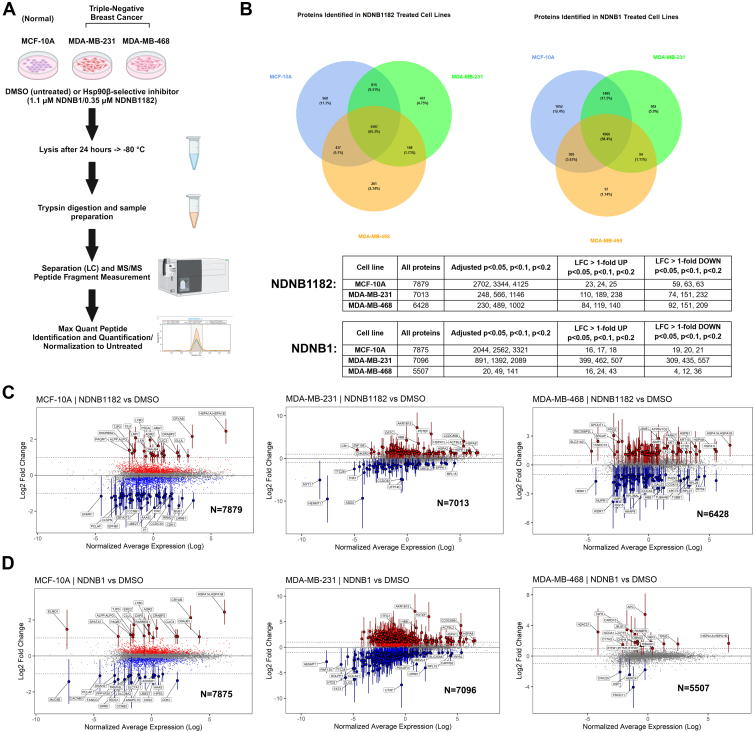
**Proteomics workflow overview and differential expression plots.***A*, proteomics workflow overview. *B*, Venn diagrams of peptides identified in each sample. Differential expression plots of MCF-10A, MDA-MB-231, or MDA-MB-468 treated with (*C*) NDNB1 or (*D*) NDNB1182 were created by plotting the average expression of each protein *versus* log_2_ fold change (LFC) between the treatment and WT. Proteins with LFC >1 and an adjusted *p* value <0.05 were highlighted with large points (up in *red*, down in *blue*), with the 95% confidence intervals plotted as error bars. Proteins with adjusted *p* value <0.05 but with an absolute LFC <1 are represented as small points, similarly colored *red* and *blue*. N = 5 biological replicates.

The proteomic changes in response to Hsp90β-selective inhibitors NDNB1 and NDNB1182 were quantified in a normalized MCF-10A and TNBC MDA-MB-231 and MDA-MB-468 cell lines by label-free quantification *via* LC–MS/MS (Fig. 2*A*). The dose of inhibitors was selected based on the concentration at which Hsp90β-dependent client proteins were degraded in the TNBC cell lines but not the MCF-10A cells (Supplemental Fig. S1); 1.1 μM NDNB1 and 0.35 μM NDNB1182. Five biological replicates with equal amounts of protein lysate from either WT (DMSO vehicle) or NDNB1/NDNB1182-treated MCF-10A, MDA-MB-231, or MDA-MB-468 cells were prepared following a 24-h treatment, which was the same as the Western blots in Supplemental Fig. S1. The lysates were digested with trypsin on silica-based filters. Following desalting, the samples were analyzed on a nanoElute 2 and timsTOF Pro 2 LC–MS/MS system with CaptiveSpray by DIA–PASEF. Two peptides were required for a positive identification, and raw files were searched using Spectronaut.

An average of 7000 proteins was positively identified across all the datasets with a false discovery rate <5%. Protein identification was reproducible with 5597 (NDNB1182) and 4966 (NDNB1) proteins being identified in all three cell lines (Fig. 2*B*). The major noted discrepancy across the data was that the MCF-10A samples had the highest number of significantly quantified proteins after the B–H correction was used to adjust the *p* values to a false discovery rate <5% (adj. *p* < 0.05). The MCF-10A samples had >2000 proteins with an adj. *p* < 0.05, meanwhile the MDA-MB-231 and MDA-MB-468 each had <1000 proteins with an adj. *p* < 0.05 (Fig. 2*B*). It was hypothesized that this was due to higher protein expression variability in the TNBC cell lines as compared with the normalized MCF-10A cells. Differential expression plots were created by plotting the average expression of each protein *versus* LFC between treated and untreated. Proteins with an LFC >1 or <−1 and an adj. *p* < 0.05 were highlighted with 95% confidence intervals plotted as error bars (Fig. 2, *C* and *D*). Standard volcano plots were generated using R and can be found in the supplemental data (Supplemental Fig. S2). In general, the MCF-10A samples showed a lower dysregulation of expression levels and a higher statistical accuracy, whereas the TNBC cell lines displayed a higher variability of expression levels and larger 95% confidence intervals. This seems to be the case as evidenced by the larger confidence intervals from five sample proteins (Supplemental Fig. S3). When requiring an absolute LFC >1 and an adj. *p* < 0.05, the NDNB1182 samples had 23, 110, and 84 proteins upregulated and 59, 74, and 92 proteins downregulated in the MCF-10A, MDA-MB-231, and MDA-MB-468 cells, respectively (Fig. 2*B*). Furthermore, the NDNB1-treated samples showed 16, 399, and 16 proteins upregulated and 19, 304, and 4 proteins downregulated in the MCF-10A, MDA-MB-231, and MDA-MB-468 cells, respectively (Fig. 2*B*). Overall, there were approximately an equal number of upregulated and downregulated proteins in each sample and more dysregulated proteins in the TNBC as compared with the MCF-10A samples. Very few proteins were measured to be statistically significant in the NDNB1-treated MDA-MB-468 samples, so most of the subsequent analysis of the MDA-MB-468 cells relied upon the NDNB1182-treated samples.

**Fig. 3:**
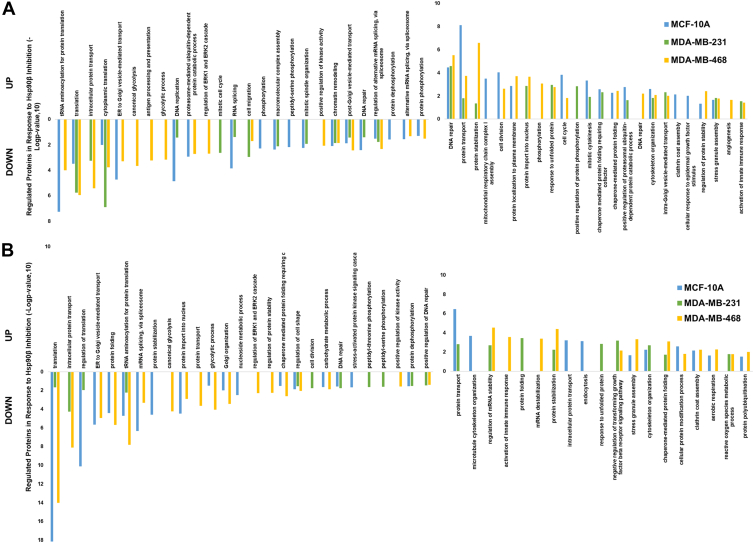
**Gene ontology biological process pathway enrichment analysis.** DAVID GO Biological Process pathway enrichment analysis for all proteins with an LFC >1 and adjusted *p* < 0.05 in (*A*) NDNB1182- or (*B*) NDNB1-treated samples. DAVID, Database for Annotation, Visualization and Integrated Discovery; GO, Gene Ontology; LFC, log_2_ fold change.

The DAVID bioinformatics database was used to perform a GO search for the enrichment analysis of GO BP of all proteins with an LFC >1 and an adj. *p* < 0.05 (Fig. 3). Proteins that were upregulated or downregulated were searched separately for each treatment and cell line. Downregulated proteins from the NDNB1182-treated samples were associated with regulation of the ERK1 and ERK2 cascade, mitotic cell cycle, phosphorylation, and antigen processing and presentation, which were expected (Fig. 3*A*) (48). Targeting Hsp90β for the disruption of kinase signaling pathways and regulation of the cell cycle has been touted as the primary mechanism of action for Hsp90β-selective inhibitors. Additional GO BPs were DNA replication, chromatin remodeling, and DNA repair. There has been evidence of DNA regulatory proteins interacting with Hsp90β, but current work is underway to investigate the specific mechanisms (49, 50, 51). GO BPs were also associated with translation, protein transport (*via* intracellular, endoplasmic reticulum to Golgi vesicle mediated, or post-Golgi vesicle mediated), and alternative mRNA splicing *via* the spliceosome. Upregulated proteins from the NDNB1182-treated samples were associated with protein stabilization, the unfolded protein response (UPR), and activation of the innate immune response (Fig. 3*A*). These were expected because of an induction of Hsp70 and potentially Hsp90α and the associated UPR. Also, Hsp90β has been shown to evade antitumor immunity, and its inhibition enhanced ICB therapy (23, 52). Other processes associated with upregulated proteins included DNA repair, protein transport, cell division, cell cycle, and DNA repair. It was unexpected that some processes associated with downregulated proteins were also associated with upregulated proteins. This could be due to a compensatory mechanism or a global downregulation of Hsp90β-regulated proteins that could be associated with both positive and negative regulation of these processes (53).

Results from the NDNB1-treated samples were synonymous albeit a few differences. For example, protein stabilization and protein folding were associated with downregulated proteins (Fig. 3*B*). Interesting GO BPs associated with upregulated proteins included regulation of mRNA stability and protein polyubiquitination. It was evident that there were contradicting trends, as proteins were upregulated or downregulated within the same pathways, so it was unclear whether the pathways were being activated or inhibited. However, many of the identified pathways have been previously associated with Hsp90 inhibition, providing support of on-target effects (24, 25). Further analysis was performed to gain an understanding of pathway activity to rectify these contradicting results.

IPA (QIAGEN) was used to assess canonical pathway activation using a B–H corrected adj. *p* < 0.05 cutoff. The resulting pathway activities were sorted by *z*-score into the top 50 pathways and then plotted as heat maps using R. One analysis was performed with all proteins with an adj. *p* < 0.05 for the MCF-10A, MDA-MB-231, and MDA-MB-468 samples (Supplemental Fig. S4). Another analysis used all proteins with an adj. *p* < 0.05 for the MCF-10A and *p* < 0.2 for the MDA-MB-231 and MDA-MB-468 samples (Supplemental Fig. S5). A higher significance threshold was used to include proteins that may be dysregulated but have a large expression variability in the TNBC as previously discussed. The following pathways were collected from both the NDNB1 and NDNB1182 samples, although the NDNB1-treated MDA-MB-468 samples were excluded because of insufficient statistically significant proteins for analysis. Interesting pathways predicted to be inhibited included cell cycle regulation, DNA repair via multiple mechanisms (base excision repair, mismatch repair, nucleotide excision repair, and homologous recombination), transcription, telomere maintenance, and Janus kinase/signal transducer and activator of transcription signaling. These results agree with the conclusions from Figure 3. Other inhibited pathways were TP53 activity, EIF2 signaling, and protein SUMOylation. Tumor suppressor TP53 activity and EIF2 signaling have been reported to be Hsp90-dependent processes and to be dependent upon Hsp90β (54, 55). Pathways predicted to be activated included the cellular response to heat stress, protein ubiquitination, microautophagy, Rho GTPase cycle, and neutrophil degranulation. This could be explained by some degree of an activation of the heat shock response and a subsequent degradation of client proteins and/or regulated proteins *via* either the canonical ubiquitin–proteasome pathway or autophagy (56, 57).

**Fig. 4:**
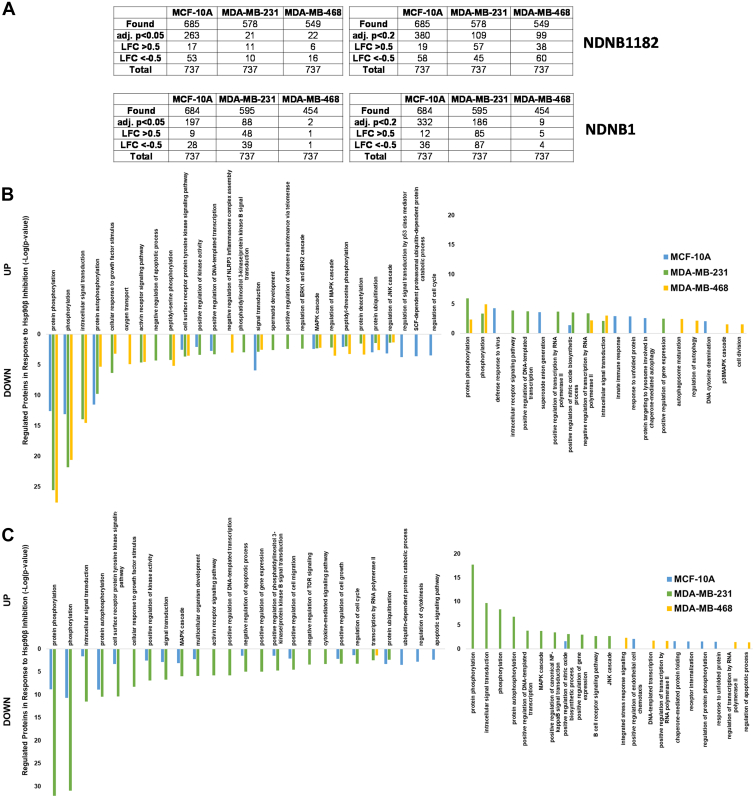
**Gene ontology search of Hsp90β interactors.***A*, Hsp90β interactors identified and dysregulated with an LFC >0.5 and an adj. *p* < 0.05 or adj. *p* < 0.2. DAVID Gene Ontology search for Biological Process pathway enrichment analysis for all previously identified Hsp90β interactors (Picard) with an LFC >0.5 and adj. *p* < 0.2 in (*B*) NDNB1182- or (*C*) NDNB1-treated samples. adj. *p*, adjusted *p* value; DAVID, Database for Annotation, Visualization and Integrated Discovery; LFC, log_2_ fold change.

The dysregulated proteins were then screened for previously identified Hsp90β interactors using The Hsp90 Chaperone Machine Database (Picard) by searching for HSP90AB1 (Hsp90β) (https://www.picard.ch/Hsp90Int (accessed 2024 07-15)). About 737 unique proteins were reported to interact with human Hsp90β. Any protein that had an LFC >0.5 and an adj. *p* < 0.2 that were previously identified to interact with human HSP90AB1 were noted as Hsp90β interactors (Fig. 4*A*).

The threshold was lowered from LFC>|1| to LFC>|0.5| to capture potential interactors exhibiting subtle dysregulation, thereby minimizing the risk of false negatives. This approach was necessary to account for the elevated variability in protein expression observed in the TNBC samples, as previously discussed. For example, in Supplemental Fig. S1, a strong degradation of previously reported beta clients, CXCR4 and c-IAP1, was not observed. NDNB1182 samples had 19, 57, and 36 upregulated and 58, 45, and 60 downregulated Hsp90β interactors in MCF-10A, MDA-MB-231, and MDA-MB-468 cells, respectively (Fig. 4*A*). NDNB1 samples had 12, 85, and 5 upregulated and 36, 87, and 4 downregulated Hsp90β interactors in MCF-10A, MDA-MB-231, and MDA-MB-468 cells, respectively. These identified and dysregulated Hsp90β interactors were subjected to the same DAVID GO BP search as previously discussed (Fig. 4, *B* and *C*). Interesting GO BPs associated with downregulated Hsp90β interactors were phosphorylation, intracellular signal transduction, cell surface receptor protein tyrosine kinase signaling pathways, protein ubiquitinylation, cell growth, cell cycle, and transcription.

GO BPs associated with upregulated Hsp90β interactors were protein phosphorylation, intracellular signal transduction, response to viral infections, innate immune response, autophagosome maturation, transcription, and protein folding. These results are consistent with previously discussed pathway enrichment and pathway activation data. Moreover, Hsp90β has been observed to play a critical role in viral infection, indicating that Hsp90β-selective inhibitors may exhibit antiviral properties (58). Specification of autophagosome maturation also supports the previously noted induction of autophagy (Fig. 3). A DAVID GO BP pathway enrichment for all 737 Hsp90β interactors can be found in the supplemental data for comparison to the results from the dysregulated interactors (Supplemental Fig. S6).

**Fig. 5:**
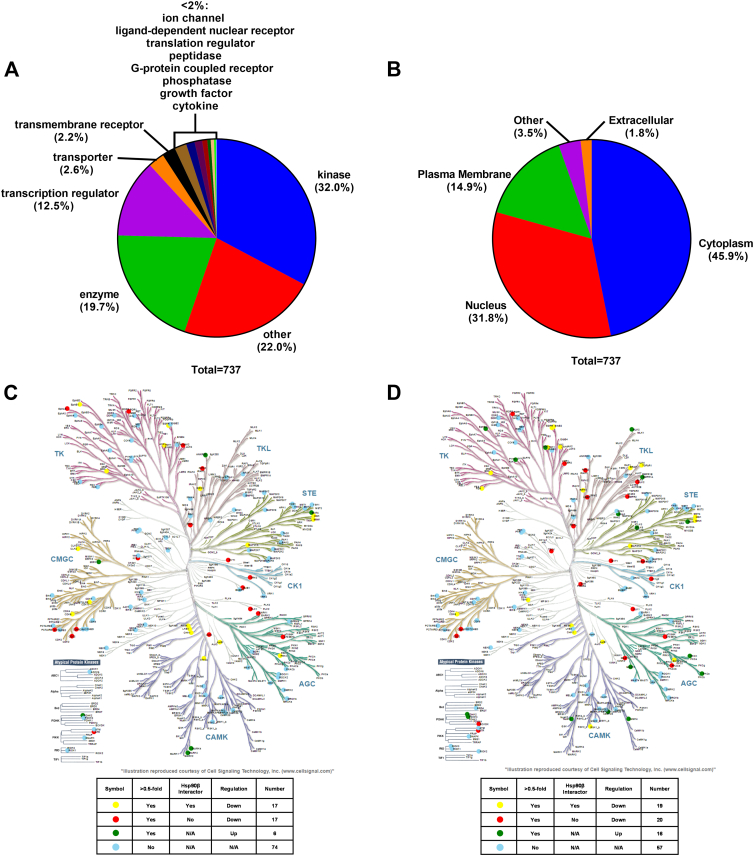
**Hsp90β interactor characterization and human kinome map.** Pie charts of the 737 Hsp90β interactors according to an IPA categorization of (*A*) protein type and (*B*) localization. KinHub human kinome map of kinase expression in response to (*C*) NDNB1182 or (*D*) NDNB1, using kinases with an LFC >0.5 in at least one cell line and an adjusted *p* < 0.05. IPA, ingenuity pathway analysis; LPC, log_2_ fold change.

The 737 total Hsp90β interactors acquired from the Picard database were further analyzed to gain an understanding of all interactors, including those not identified or dysregulated in the proteomics data. IPA (Qiagen) was used to collect information about the 737 proteins, including the protein type and subcellular location (Fig. 5, *A* and *B*). About 32.0% of the 737 interactors are “kinases,” highlighting the rationale for targeting multiple signaling pathways *via* a single molecular target *via* Hsp90 inhibition (Supplemental Table S1) (57). In addition, “other” encompassed 22.0% of the interactors, “enzyme” 19.7%, and “transcription regulator” 12.5%. These statistics exemplify the diverse functions that are regulated by Hsp90 and more specifically, Hsp90β.

In terms of location, 45.9% are cytosolic, 31.8% are nuclear, and 14.9% are located at the plasma membrane (Supplemental Table S2). A high proportion of cytosolic interactors is expected, as Hsp90β is one of the two cytosolic Hsp90 isoforms. However, the abundance of nuclear proteins emphasizes Hsp90β′s role in the regulation of DNA repair, DNA replication, chromatin remodeling, and transcription, which are both the prior pathway enrichment/activity data and the literature support.

Effects on the kinome were investigated by mapping all the significantly dysregulated (LFC >0.5, adj. *p* < 0.05) kinases onto the human phylogenetic kinome tree using KinHub (Fig. 5). There were 34 downregulated kinases with 17 identified as Hsp90β interactors when compared with only six upregulated kinases in the NDNB1182 samples. In the NDNB1 samples, there were 39 downregulated kinases with 19 identified as Hsp90β interactors as compared with only 16 upregulated kinases. A similar map of all reported kinase Hsp90β interactors can be found in the supplemental data (Supplemental Fig. S7). Approximately half of all downregulated kinases were Hsp90β interactors, which is unsurprising, as they account for 236 of the 538 human kinases (Supplemental Table S1). The other downregulated kinases that are not Hsp90β interactors may contain undiscovered kinase interactors or simply kinases that are regulated by Hsp90β.

**Fig. 6:**
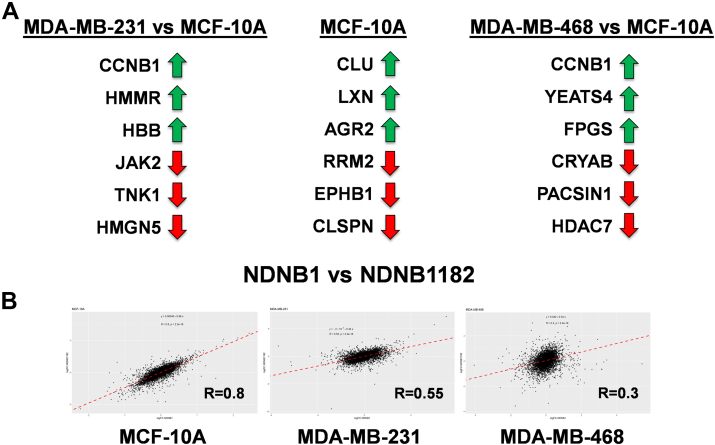
**Cell line comparison analysis of dysregulated proteins and comparison of NDNB1 vs NDNB1182.***A*, example of dysregulated proteins from NDNB1182 cell line comparison analysis. *B*, Pearson's correlation coefficients of NDNB1 *versus* NDNB1182 in MCF-10A, MDA-MB-231, and MDA-MB-468 samples.

A comparison between cell lines for each treatment was performed using B–H-adjusted *t* tests to understand whether there were any cell line–specific trends/proteins dysregulated. Volcano plots of the relative LFC differences in response to NDNB1 and scatter plots were generated with proteins that had an LFC difference >1 highlighted (Supplemental Fig. S8). Similar plots were generated for NDNB1182 (Supplemental Fig. S9). Predominately, a majority of the highest dysregulated proteins were common to both the TNBC and TNBC *versus* MCF-10A datasets with comparable LFC levels. These results indicate that the proteins affected in the TNBC cells are less dysregulated in the normalized MCF-10A cells. Therefore, although similar pathways are inhibited/activated in the MCF-10A and TNBC cells (Supplemental Figs. S4 and S5), the Hsp90β-selective inhibitors appear to exhibit selectivity for cancer cell lines. However, there were still interesting examples of differentially regulated proteins. Tyrosine-protein kinase JAK2, which is an Hsp90β interactor, was significantly more downregulated in the NDNB1182-treated MDA-MB-231 *versus* MCF-10A samples (Fig. 6*A*). In the same data set, nonreceptor tyrosine-protein kinase TNK1, which is also an Hsp90β interactor, was also significantly more downregulated. Another Hsp90β interactor, Clusterin (CLU), was not dysregulated in the MDA-MB-231 cell line but significantly upregulated in the MCF-10A. Interestingly, non-Hsp90β interactor G2/mitotic-specific cyclin-B1 (CCNB1) was upregulated in the MDA-MB-231 *versus* MCF-10A samples. This could be a compensatory mechanism in response to cell cycle arrest, as a similar upregulation of CCNB1 has been observed in response to doxorubicin-induced G2/M arrest (59). CCNB1 was also upregulated in the NDNB1182-treated MDA-MB-468 *versus* MCF-10A samples. Alpha-crystallin B chain (CRYAB) was downregulated in the MDA-MB-468 *versus* upregulated in the MCF-10A. CRYAB activates immune checkpoints and inhibits apoptosis and is worthy of subsequent investigation (60). Another protein downregulated *versus* upregulated in the same samples was protein kinase C and casein kinase substrate in neurons 1 (PACSIN1). Deficiency of PACSIN1 enhances chemosensitivity of gastric cancer to ICB therapy and enhances antigen presentation (61). Both the previous two examples could provide further rationale as to how Hsp90β-selective inhibitors sensitize cancer to ICB.

A similar comparison analysis was performed to compare NDNB1 *versus* NDNB1182 in each cell line using B–H-adjusted *t* tests (Supplemental Fig. S10). In addition, Pearson's correlation coefficients were calculated using R, which were *R* = 0.8, *R* = 0.55, and *R* = 0.3 for the MCF-10A, MDA-MB-231, and MDA-MB-468 samples, respectively (Fig. 6*B*/Supplemental Fig. S11). Responses in the normalized MCF-10A samples were consistent between NDNB1 and NDNB1182, which is likely because of a small variance in dysregulation. In contrast, the response was somewhat consistent in the MDA-MB-231 samples and inconsistent in the MDA-MB-468 samples. The discrepancy in the MDA-MB-468 response is likely because of the relatively few statistically significant proteins identified in the NDNB1-treated MDA-MB-468 samples (Fig. 2*B*).

**Fig. 7:**
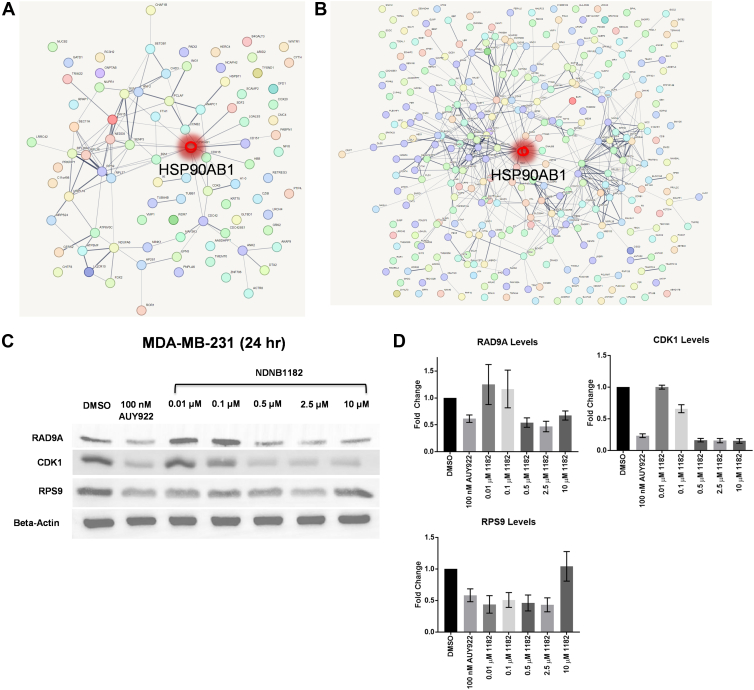
**Protein interaction network and expression of key Hsp90β-regulated proteins.** Search Tool for the Retrieval of Interacting Genes/Proteins (STRING) database map to visualize protein interactions with LFC <−1 and adjusted *p* < 0.05 for (*A*) NDNB1182 against MDA-MB-468 and (*B*) NDNB1 against MDA-MB-231. Hsp90β (HSP90AB1) is highlighted in *red*. *C*, Western blot of MDA-MB-231 cells treated with NDNB1182 for 24 h. *D*, densitometry of RAD9A, CDK1, and RPS9 levels reported as the average ± SD from N = 3 biological replicates. LFC, log_2_ fold change.

The STRING database was used to map HSP90AB1 (Hsp90β) along with all proteins with an LFC <−1 and an adj. *p* < 0.05 for each inhibitor and each cell line sample. NDNB1182 against MDA-MB-468 was emphasized, since it was the reliable MDA-MB-468 sample, which displayed a modest protein interaction network centered around HSP90AB1 (Fig. 7*A*). A similar, but much more complex, interaction map can be observed in the NDNB1-treated MDA-MB-231 sample (Fig. 7*B*). STRING maps for the remaining samples can be found in the supplemental data (Supplemental Fig. S12). Conclusions from the two prioritized maps are that most of the downregulated proteins in the TNBC samples appear to be centrally involved with Hsp90β directly or Hsp90β-interacting proteins.

In addition, the interaction maps allowed the identification of new proteins that are central “nodes” that appear to interact with several other downregulated proteins that were not previously known Hsp90β interactors. As such, they should be investigated as potential Hsp90β interactors or as potential key Hsp90β-regulated proteins. For a few examples, CDK1 was noted as a downregulated “node” in several of the protein interaction maps. CDK1 has been reported as an Hsp90α interactor that was observed to be degraded in response to Hsp90 inhibition, and there is evidence that it may be a valuable CDK target in breast cancer (62, 63). In contrast to targeting the G_1_/S transition through degradation of CDK4/6, CDK1 degradation induces G_2_/M arrest. In addition, CDK1 binds to CCNB1 to promote cell cycle progression, which was differentially upregulated in the TNBC as discussed earlier, warranting further investigation. Cell cycle checkpoint control protein RAD9A has been implicated in the DNA damage checkpoint pathway and DNA repair *via* homologous recombination (64). Myeloid differentiation factor 88 (MYD88) plays a critical role in breast cancer through diverse functions. Two examples include activation of PI3K/Akt and Toll-like receptor/NF-κB pathways to modulate the immune response (*e.g.*, *via* cytokine secretion) and regulation of cancer stem cell renewal (65). However, low ribosomal protein S9 (RPS9) expression, which was identified as a downregulated “node,” is associated with poor survival in breast cancer patients (66). Furthermore, RPS6 and several ribosomal protein S6 kinases (RPS6Ks) are reported Hsp90β interactors (67, 68).

RAD9A, CDK1, and RPS9 were selected for further evaluation. MDA-MB-231 cells were treated with NDNB1182 or Hsp90 pan-inhibitor AUY922 for 24 h and analyzed by Western blot (Fig. 7*C*). RAD9A levels decreased dose-dependently upon Hsp90β inhibition, indicating that RAD9A is stabilized or regulated by Hsp90β. Whether this regulation is direct, which would designate RAD9A as a client protein, or indirect, through another Hsp90β-regulated process, remains undetermined. The same rationale could be applied to CDK1, which was also downregulated in response to Hsp90β inhibition. Since CDK1 has previously been reported as an Hsp90α interactor and is degraded in response to Hsp90 pan-inhibition, these results suggest that CDK1 is a client of Hsp90β. RPS9 levels also decreased, so RPS9 is also regulated by Hsp90β and likely an Hsp90β interactor.

## Discussion

Hsp90β-selective inhibitors have been shown to synergize with ICB therapy and maintain a favorable safety profile, indicating they could be a promising candidate for an anticancer therapeutic. The purpose of this study was to gain a mechanistic understanding of how Hsp90β-selective inhibitors exert anticancer effects and determine whether they manifest selectivity for cancer cells. Hsp90β-selective inhibitors NDNB1 and NDNB1182 exhibit low or submicromolar antiproliferative IC_50_ values against TNBC cell lines with a 2-5-fold selectivity for cancer cells. Inhibition of Hsp90β results in the degradation of Hsp90β-dependent client proteins, such as CDK4, CDK6, CXCR4, and c-IAP1, among others. In addition, selective inhibition of Hsp90β over Hsp90α avoids induction of the heat shock response, at least in part, although Hsp70 levels are still upregulated.

Herein, the effects of two Hsp90β-selective inhibitors, NDNB1 and NDNB1182, on the proteome were investigated against an MCF-10A normalized breast cell line and two TNBC cell lines, MDA-MB-231 and MDA-MB-468. Fewer proteins were measured as statistically significant in the TNBC samples because of a larger variability of expression in the cancers as compared with the MCF-10A samples. However, there were significantly more dysregulated (LFC >1) proteins in the TNBC with roughly an equal number of upregulated and downregulated proteins. Previous proteomic analyses of Hsp90 pan-inhibitors found that dysregulated proteins were predominantly downregulated in response to Hsp90 inhibition. This contrasting difference suggests that Hsp90β inhibition results in an imbalance in cellular processes rather than shear degradation of Hsp90-dependent clients. In fact, this hypothesis is supported by the results of GO BP pathway enrichment and IPA canonical pathway activation analyses. The results show that proteins that were downregulated and upregulated were associated with overlapping cellular pathways with conflicting trends. However, IPA pathway activation showed that overall, many of the identified pathways were inhibited. This could be due to a compensatory mechanism, or Hsp90β-regulated proteins are associated with both positive and negative regulation of these processes.

Some of the identified effects were expected such as downregulation and inhibition of kinases and kinase signaling pathways, cell cycle, DNA repair, and regulation of transcription, to name a few. Others included protein transport, translation, and chromatin remodeling. Upregulated processes included protein stabilization, the UPR, and activation of the innate immune response. Focusing on previously identified Hsp90β interactors revealed that these processes were enriched in Hsp90β interactors. In fact, Hsp90β interactors are associated with a variety of biological functions, which is exemplified by their array of protein type and localization, although most interactors are kinases and localized to the cytosol or nucleus. Furthermore, most of the dysregulated proteins were not previously identified as Hsp90β interactors, indicating that Hsp90β could regulate up to 1000 proteins, which are not necessarily Hsp90β clients.

Hsp90β and its interactors regulate housekeeping processes essential for cell development and proliferation. Unsurprisingly, signaling proteins (receptors, kinases) and proteins responsible for regulation of DNA (transcription factors, DNA repair, and chromatin remodeling) were emphasized in the identified and dysregulated proteins (69, 70). This was expected, as they encompass essential processes for cell survival and include attractive drug targets to inhibit cancer growth. In addition, CRYAB and PACSIN1 were identified and provide rationale for how Hsp90β inhibition enhances ICB therapy. Other interesting proteins that were downregulated included ubiquitin protein ligases SIAH1, DTX2, ANAPC1, and HERC4 as well as ubiquitin-conjugating enzymes UBE2T, UBE2L5, and UBE2S. Many of these were not previously identified Hsp90β interactors, but they may provide a mechanistic explanation for Hsp90β-dependent protein ubiquitinylation.

A STRING database search revealed that Hsp90β is a central interactor with downregulated proteins. The interaction networks identified “node” proteins that were not previously identified as Hsp90β interactors but may be regulated by Hsp90β or are previously unidentified Hsp90β clients. Further evaluation showed that RAD9A expression is regulated by Hsp90β, identifying a previously unknown Hsp90β-dependent DNA repair mechanism. Hsp90β also regulated CDK1 expression, which provides rationale for an additional cell cycle arrest mechanism. Future directions will investigate whether RAD9A and CDK1 are client substrates of Hsp90β.

A cell line comparison analysis and comparison between NDNB1 and NDNB1182 in each cell line was performed. The results support that target proteins are more dysregulated in the TNBC cells lines as compared with the normalized MCF-10A cells, which is additional evidence for a selectivity for cancer cells. It is important to note that the effects observed in the TNBC cell lines were similarly observed in the normalized MCF-10A cells although to a lesser extent. This indicates that Hsp90β-selective inhibitors could inhibit housekeeping processes essential for cell development and proliferation in normal tissues. However, the active Hsp90 heteroprotein complex found in cancer exhibits ∼200-fold higher affinity for ATP than the Hsp90 homodimer found in normal tissue, so ATP-competitive inhibitors accumulate in cancer over normal tissues (14, 19).

NDNB1 and NDNB1182 were expected to exhibit the same mode of action, especially because the doses were adjusted for differences in potency. As the proteomics methodology was reliable, the differences between the responses in the TNBC samples may be due to the different concentrations of inhibitor. A minimal concentration of NDNB1/NDNB1182 was chosen to avoid potential inhibition of Hsp90α at higher doses. At concentrations wherein strong client degradation is not observed, there may be a greater variability of response. This has recently been documented to occur in response to Hsp90 pan-inhibitors (71). With regard to Hsp90β inhibition, this could mechanistically be rationalized by Hsp90α partially compensating for Hsp90β functionally and/or a moderate induction of Hsp90α.

Overall, the quantitative proteomics approach used in this study employed strict filters to ensure statistical accuracy, including >2 peptides to be detected for protein identification, two allowed missed cleavages, and a false discovery rate of 0.01 at the peptide and protein levels. The use of five biological replicates and three technical replicates guaranteed reproducibility of the measurements. As a result, 20,598 entries were collected, and an average of 6966 proteins were quantified. The bottom–up LC–MS/MS proteomics approach provided an accurate and broad investigation of the cellular functions effected by Hsp90β-selective inhibition. However, there were limitations to the study. Common disadvantages of a label-free approach include higher variability between injections and difficulty in detecting low-abundance proteins. For example, the NDNB1-treated MDA-MB-468 had few statistically significantly dysregulated proteins measured, which was likely because of high variation across injections or possibly because of high variability across biological replicates because of an inconsistent response. This sample was largely omitted from useful result interpretation for this reason. In addition, only two similar cancer cell lines were used, which does not allow an understanding of the inhibitor effects on distinct TNBC subtypes. The study design and accuracy of the changes in protein levels were still useful to understanding how Hsp90β-selective inhibitors manifest anticancer effects against TNBC.

In conclusion, this is the first proteomic analysis of Hsp90β-selective inhibitors. The results show that Hsp90β-selective inhibitors NDNB1 and NDNB1182 exhibit a selectivity for TNBC over normalized MCF-10A cells. Potential mechanisms of action underlying the anticancer effects of NDNB1 and NDNB1182 are proposed. Namely, kinases and associated cell signaling pathways, cell cycle proteins, and DNA repair are primarily dependent upon Hsp90β. In addition, RAD9A, CDK1, and RPS9 were identified as potential Hsp90β client substrates, highlighting the use of proteomics to identify other previously unknown clients, Hsp90β interactors, or Hsp90β-regulated proteins. The three example proteins selected exemplify a mechanistic explanation for inhibition of DNA-repair (RAD9A), cell cycle (CDK1), and translation (RPS9) as a result of Hsp90β inhibition.

## Data Availability

All data generated or analyzed during this study are included in this published article (and its supplemental data files). Raw files can be found on MassIVE with identifier MSV00096357 (ftp://MSV00096357@massive.ucsd.edu) and Proteome Exchange PXD (PXD057678). Password is: HSP902024TR!01. The full scripts for protein identification and statistical analysis can be found on GitHub (https://github.com/Champion-Lab/HSP90Binh).

## Supplemental Data

This article contains supplemental data.

## Conflict of interest

S.J.M. and B.S.J.B. are equity holders at the Grannus Therapeutics, Inc, a company that aims to bring Hsp90b-selective inhibitors to the clinic.
