# Supplementary material for: Proteomic Analysis of Hsp90β-Selective Inhibitors Against Triple-Negative Breast Cancer to Gain a Mechanistic Insight
Source: Mol Cell Proteomics. 2025 Jul 24;24(9):101043. doi: 10.1016/j.mcpro.2025.101043 (PMC12446539; doi:10.1016/j.mcpro.2025.101043)
Supplement: Supplementary Data 16 [file mmc16.docx]

**Supporting Information for**

**Proteomic Analysis of Hsp90β-selective Inhibitors Against Triple-negative Breast Cancer (TNBC) to Gain a Mechanistic Insight**

**Tyelor S. Reynolds^[1]^, Daniel Hu^[2]^, Simon Weaver^[2]^, Emma C. Ronck^[1]^, Sanket J. Mishra^[3]^, Matthew M. Champion^[2]^, and Brian S. J. Blagg^[1]*^**

^1^Department of Chemistry and Biochemistry, The University of Notre Dame, 305 McCourtney Hall, Notre Dame, IN, 46556, USA, E-mail: bblagg@nd.edu

^2^Department of Chemistry and Biochemistry, The University of Notre Dame, 153 McCourtney Hall, Notre Dame, IN, 46556, USA, E-mail: mchampio@nd.edu

^3^Grannus Therapeutics Inc., 1400 E Angela Blvd, South Bend, IN, US 46617

*Corresponding author

**Running Title: Proteomic Analysis of Hsp90β Inhibition**

**Table of Contents Page no.**

**Western Blots (Figure S1)**....….……………………………………………………………………...S2

**Volcano Plots (Figure S2)**....….……………………………………………………………………...S3

**Protein Expression Variance (Figure S3)**..………………………………………………………...S3

**IPA Pathway Heat Maps, adj. p<0.05 (Figure S4)**.…………………………………………………S4

**IPA Pathway Heat Maps, adj. p<0.2 (Figure S5)**.…………………………………………………..S5

**Hsp90β Interactors GO BP (Figure S6)**.…………………………………………………………….S6

**Hsp90β Interactors Protein Type (Table S1)**.………………………………………………………S6

**Hsp90β Interactors Protein Localization (Table S2)**……………………………………………...S6

**Hsp90β Interactors Kinome Map (Figure S7)**……………………………………………………...S7

**NDNB1 Cell Line Differential Expression Comparison (Figure S8)**…...…………………….S8

**NDNB1182 Cell Line Differential Expression Comparison (Figure S9)**…...………….….….S9

**NDNB1 vs NDNB1182 Differential Expression Comparison (Figure S10)**…..……….….…..S10

**NDNB1 vs NDNB1182 Correlation Plots (Figure S11)**…..……….….…..................................S11

**STRING Interaction Maps (Figure S12)**…..……….….….........................................................S12

**Figure S1**. Western blot analysis to detect inhibition of Hsp90β in MCF-10A, MDA-MB-231, and MDA-MB-468 cells after 24-hr treatment with A) **NDNB1** or B) **NDNB1182**. 0.25% DMSO (vehicle) and 100 nM AUY922 (Hsp90 pan-inhibitor) used as controls.

**Figure S2**. Volcano plots were generated in R by plotting the significance (-10log(p-value)) of each protein vs log_2_ fold change (LFC) between the wild type (DMSO) and A) NDNB1182 or B) NDNB1 treated samples. Proteins with LFC greater than 1 and an adjusted p-value <0.05 were highlighted (up in red, down in blue). N=5 biological replicates.

**Figure S3**. Protein expression variance to compare statistical confidence across the three cell lines in the **NDNB1182** treated samples. Five dysregulated proteins were selected and compared by the difference between the Log fold change and one-sided confidence interval.

**Figure S4**. Heat map of canonical pathway activation from Ingenuity Pathway Analysis. Using all proteins with an adj. p<0.05 for the MCF-10A, MDA-MB-231, and MDA-MB-468 samples in response to A) **NDNB1182** or B) **NDNB1**.

**Figure S5**. Heat map of canonical pathway activation from Ingenuity Pathway Analysis. Using all proteins with an adj. p<0.05 for the MCF-10A and p<0.2 for the MDA-MB-231 and MDA-MB-468 samples in response to A) **NDNB1182** or B) **NDNB1**.

**Figure S6**. Analysis of all 737 previously identified Hsp90β interactors (Picard) via DAVID Gene Ontology (GO) search for biological processes (BP) pathway enrichment.

| **Type** | **Counts** | **%** |
| --- | --- | --- |
| kinase | 236 | 32.0 |
| other | 162 | 22.0 |
| enzyme | 145 | 19.7 |
| transcription regulator | 92 | 12.5 |
| transporter | 19 | 2.6 |
| transmembrane receptor | 16 | 2.2 |
| ion channel | 14 | 1.9 |
| ligand-dependent nuclear receptor | 10 | 1.4 |
| translation regulator | 9 | 1.2 |
| peptidase | 6 | 0.8 |
| G-protein coupled receptor | 4 | 0.5 |
| phosphatase | 4 | 0.5 |
| growth factor | 2 | 0.3 |
| cytokine | 1 | 0.1 |
| **Total** | **737** |  |

**Table S1**. Analysis of all 737 previously identified Hsp90β interactors (Picard). Count of protein type acquired from Ingenuity Pathway Analysis.

| **Location** | **Counts** | **%** |
| --- | --- | --- |
| Cytoplasm | 338 | 45.9 |
| Nucleus | 234 | 31.8 |
| Plasma Membrane | 110 | 14.9 |
| Other | 26 | 3.5 |
| Extracellular | 13 | 1.8 |
| **Total** | **737** |  |

**Table S2**. Analysis of all 737 previously identified Hsp90β interactors (Picard). Count of protein localization acquired from Ingenuity Pathway Analysis.

**Figure S7**. KinHub human kinome map of all kinase Hsp90β interactors, even those not identified in the proteomics data sets.

**Figure S8**. The LFC between **NDNB1** and WT for A) MDA-MB-231 vs MCF-10A, B) MDA-MB-468 vs MCF-10A, or C) MDA-MB-468 vs MDA-MB-231 which were used to perform a meta differential expression between cell lines, using t-tests. Volcano plots were created to show the change in LFC and significance for each treatment between cell lines. The dashed lines represent 1 LFC difference, and the red line represents the significance cutoff after the t-test.

**Figure S9**. The LFC between **NDNB1182** and WT for A) MDA-MB-231 vs MCF-10A, B) MDA-MB-468 vs MCF-10A, or C) MDA-MB-468 vs MDA-MB-231 which were used to perform a meta differential expression between cell lines, using t-tests. Volcano plots were created to show the change in LFC and significance for each treatment between cell lines. The dashed lines represent 1 LFC difference, and the red line represents the significance cutoff after the t-test.

**Figure S10**. The LFC between **NDNB1182** and **NDNB1** for A) MCF-10A, B) MDA-MB-231, or C) MDA-MB-468, which were used to perform a meta differential expression between cell lines, using t-tests. Volcano plots were created to show the change in LFC and significance for each treatment between cell lines. The dashed lines represent 1 LFC difference, and the red line represents the significance cutoff after the t-test.

**Figure S11**. The LFC between **NDNB1182** and **NDNB1** for A) MCF-10A, B) MDA-MB-231, or C) MDA-MB-468 from to Figure S7 was used to generate correlation plots and determine the Pearson correlation coefficients.

**Figure S12**. Search Tool for the Retrieval of Interacting Genes/Proteins (STRING) database map to visualize protein interactions with LFC less than -1 and adj. p<0.05 for **NDNB1182** against A) MCF-10A or B) MDA-MB-231, or **NDNB1** against C) MCF-10A or D) MDA-MB-468. Hsp90β (HSP90AB1) is highlighted in red.
